# Sprint Interval Running and Continuous Running Produce Training Specific Adaptations, Despite a Similar Improvement of Aerobic Endurance Capacity—A Randomized Trial of Healthy Adults

**DOI:** 10.3390/ijerph17113865

**Published:** 2020-05-29

**Authors:** Sigbjørn Litleskare, Eystein Enoksen, Marit Sandvei, Line Støen, Trine Stensrud, Egil Johansen, Jørgen Jensen

**Affiliations:** 1 Department of Physical Performance, Norwegian School of Sport Sciences, 0863 Oslo, Norway; sigbjorn.litleskare@inn.no (S.L.); eystein.enoksen@nih.no (E.E.); maritsandvei@hotmail.com (M.S.); linemor@gmail.com (L.S.); trine.stensrud@nih.no (T.S.); e.i.johansen@nih.no (E.J.); 2 Department of Sports and Physical Education, Inland Norway University of Applied Sciences, 2406 Elverum, Norway

**Keywords:** maximal oxygen consumption, heart rate, oxygen pulse, shuttle run, repeated sprint ability

## Abstract

The purpose of the present study was to investigate training-specific adaptations to eight weeks of moderate intensity continuous training (CT) and sprint interval training (SIT). Young healthy subjects (*n* = 25; 9 males and 16 females) performed either continuous training (30–60 min, 70–80% peak heart rate) or sprint interval training (5–10 near maximal 30 s sprints, 3 min recovery) three times per week for eight weeks. Maximal oxygen consumption, 20 m shuttle run test and 5·60 m sprint test were performed before and after the intervention. Furthermore, heart rate, oxygen pulse, respiratory exchange ratio, lactate and running economy were assessed at five submaximal intensities, before and after the training interventions. Maximal oxygen uptake increased after CT (before: 47.9 ± 1.5; after: 49.7 ± 1.5 mL·kg^−1^·min^−1^, *p* < 0.05) and SIT (before: 50.5 ± 1.6; after: 53.3 ± 1.5 mL·kg^−1^·min^−1^, *p* < 0.01), with no statistically significant differences between groups. Both groups increased 20 m shuttle run performance and 60 m sprint performance, but SIT performed better than CT at the 4th and 5th 60 m sprint after the intervention (*p* < 0.05). At submaximal intensities, CT, but not SIT, reduced heart rate (*p* < 0.05), whereas lactate decreased in both groups. In conclusion, both groups demonstrated similar improvements of several performance measures including VO_2max_, but sprint performance was better after SIT, and CT caused training-specific adaptations at submaximal intensities.

## 1. Introduction

Manipulation of duration and intensity of exercise bouts change the demands of metabolic pathways within muscle cells, as well as oxygen delivery to exercising muscles [1]. The training adaptations that occur after repeated bouts of exercise are to some degree specific to that particular exercise [1,2], but both high intensity interval training and continuous training bouts increase VO_2max_ and oxidative capacity in skeletal muscles [1,2,3]. Within this context, it is of interest to clarify the specific adaptations of different training protocols to optimize endurance training, health and performance. 

There has recently been a lot of interest in a type of high intensity interval training known as sprint interval training (SIT). SIT is (often) performed as 30 s of “all out” sprints with 2.5–4.5 min of rest between sprints [4,5,6]. Several cycling studies have reported that this type of training improves maximal oxygen consumption (VO_2max_), endurance performance and the oxidative capacity of skeletal muscle [3,4,5,6,7,8,9,10,11,12]. Previous studies have also demonstrated that the magnitude of improvement in endurance performance and VO_2max_ after SIT is comparable to continuous cycling at moderate intensity [3,4]. Furthermore, research also suggest that SIT is an efficient approach to improve several important health parameters in addition to VO_2max_, such as insulin sensitivity, blood pressure, cardiovascular function, and body composition [13].

Because most previous studies on SIT adaptations have used a cycling protocol, there is limited knowledge about sprint interval running [14]. This is unfortunate, as running is a basic and popular type of exercise. More importantly, there are several fundamental differences between cycling and running exercise. Power output during sprint exercise is substantially higher in cycling than in running [15]. There are also several physiological differences, such as higher heart rate (HR), higher fat oxidation and higher muscle mass activation in running than in cycling [16,17]. Thus, results from sprint interval cycling may not be directly applicable to sprint interval running [18].

Only a few previous studies have investigated the effects of sprint interval running. In most of these studies, SIT is added to the training program of trained endurance athletes [19,20,21]. However, one previous study has compared the effect of sprint interval and traditional endurance running in healthy untrained subjects [22]. Macpherson et al. [22] reported similar improvements of VO_2max_ and endurance performance after SIT and continuous running at moderate intensity. Interestingly, VO_2max_ improved in the SIT group without affecting cardiac output, whereas continuous running increased cardiac output, as expected. The study by Macpherson et al. [22] revealed that sprint interval running and continuous running produced similar improvements of aerobic performance, but still caused training-specific physiological adaptations. Because there is limited data available on this topic, it is of great interest to investigate training-specific adaptations of sprint interval running and continuous running. 

The purpose of this study was therefore to compare performance and health related adaptations of continuous training (CT) and SIT, performed as running, on VO_2max_, 20 m shuttle run performance, repeated sprint ability (RSA) and the physiological response to submaximal exercise. We hypothesized that both types of training would improve VO_2max_ and 20 m shuttle run similarly, and that training-specific adaptations would occur at submaximal exercise in favor of CT and during RSA in favor of SIT.

## 2. Materials and Methods 

### 2.1. Participants

Participants were recruited through the official webpage of the Norwegian School of Sport Sciences, and printed and electronic flyers posted in various places in the local area of northern Oslo and in social media, respectively. Forty-eight subjects volunteered and were screened for participation. The inclusion criteria for participation were: (1) non-smokers; (2) body mass index (BMI) < 30 kg·m^−2^; (3) no cardiovascular or metabolic disease; (4) no systematic endurance training during the last two years (≤2 sessions per week). Twenty-nine subjects met these criteria and were invited to participate. Subjects were matched based on gender and VO_2max_, and then randomly assigned by coin toss to either CT or SIT. Four subjects dropped out during the training intervention; One dropped out during week 1 due to receiving a job offer (CT, male 21 years), one, during week 2, after realizing that participation in the intervention was not compatible with his life situation (SIT, male, 21 years), one during week 5, due to unspecified reasons (CT, male, 22 years), and one during week 8, due to moving to a different region (SIT, female, 22 years). Thus, 25 subjects (9 males and 16 females) completed the training intervention.

### 2.2. Training Protocol 

Both groups completed eight weeks of training. Each week consisted of three training sessions, separated by at least one resting day. Training sessions were organized and supervised by qualified instructors. Subjects were occasionally allowed to perform sessions at home if participation in organized sessions was problematic. The training intensity was controlled during all sessions by heart rate monitors (Polar Sport Tester RS800CX, Polar Electro, OY, Kempele, Finland). An adherence of >85% (19 of 24 training sessions, including sessions performed at home) was required. Subjects were instructed to maintain their normal diet and lifestyle throughout the intervention.

The CT group was instructed to maintain an intensity corresponding to 70–80% HR_peak_ at all training sessions. Organized training sessions were performed on slightly undulating terrain. During the first week, the CT group performed 30 min of running. The time then increased by five minutes per week, up to a total of 60 min. The SIT group consisted of 30 s sprints at near maximal effort, with three minutes of rest between each sprint. The training intensity of SIT sessions was evaluated subjectively during sessions, while the HR data was used to verify that the individual participant did not have a session or interval that deviated from their usual level of effort. During the first week, the SIT group performed five sprints per session. The number of sprints then increased gradually, until a total of 10 sprints per session in weeks 7 and 8. When the number of sprints reached seven, subjects were given six minutes of rest midway through the training session. All sprints were performed on slightly uphill terrain. Prior to all training sessions, the CT group performed a ten-minute warm-up at an intensity corresponding to 60–75% of HR_peak_. The SIT group performed a ten-minute warm-up at an intensity corresponding to 60–85% of HR_peak_, followed by three incremental strides of about 80 m. After each training session, all subjects performed five minutes of walking or running at intensities below 70% of HR_peak_. The training volume in CT and SIT was not matched.

### 2.3. Measures

Incremental treadmill test to exhaustion. The test was performed on a motorized treadmill (Woodway pps55 sport, Woodway Gmbh, Weil an Rhein, Germany). Oxygen consumption (VO_2_) was measured through a 2-way mouthpiece (Hans Rudolph Instr., Shawnee, KS, USA) and a sling, which was connected to an O_2_ and CO_2_ analyzer (Oxycon Champion, Jaeger Instruments, Hoechberg, Germany). Samples of O_2_ and CO_2_ were collected continuously from a mixing chamber, with average values obtained over 30-s intervals. The gas analyzer was calibrated before each test with ventilated indoor air and standardized gas concentrations, to span the concentration range observed during exercise. The expired volume was measured with a turbine (Triple V volume transducer, Leipzig, Germany), and volume calibration was performed regularly with a 3-L syringe. 

The incremental test to exhaustion followed current recommendations for test duration [23], and was performed according to the standard protocol of the Norwegian Olympic Sports Centre (see e.g., [24]). Prior to the pre-test, subjects performed two familiarization tests to reduce the learning effect, following the recommendations of Edgett et al. [25]. Identical procedures were conducted for familiarization, pre- and post-test. All subjects performed a 15-min warm-up of gradually increasing intensity. The last five minutes of the warm-up were performed with an inclination of 5.3%, as was the incremental test. The starting speed was chosen in order to exhaust the subjects after ~5 min. Running speed was initially increased by 1 km·h^−1^ every minute. At the end of the test, running speed was either maintained or increased by 0.5 km·h^−1^, to allow at least one minute running at the final speed. VO_2max_ was determined as the average of the highest values achieved over two subsequent 30-s measurements. Verbal encouragement was given throughout the test. Two minutes after completion, a capillary blood sample was obtained and 20 µl of blood was injected into a lactate analyzer (1500 SPORT, YSI Inc., Yellow Springs Instr., Yellow spring, OH, USA), with the help of a standard injector. The lactate analyzer was calibrated before each test with a 5.0 mM lactate standard. The main criterion for evaluating whether VO_2max_ was achieved was a plateau in oxygen consumption. A levelling-off of the VO_2_ curve was used in conjunction with a lactate value ≥ 6 mmol·l^−1^ and respiratory exchange ratio (RER) > 1.10 as secondary criteria. HR was monitored throughout the test (Polar Sport Tester RS800CX, Polar Electro, OY, Kempele, Finland) and the highest value achieved was defined as HR_peak_. 

Submaximal treadmill test. The submaximal treadmill test was conducted with the same equipment as described above and consisted of four stages of five minutes on a motorized treadmill. The running speed at each stage was individualized based on each subject’s VO_2max_ and a general relationship between running speed and VO_2_. This relationship was estimated based on data from a pilot study. The purpose of the procedure was to establish four individualized stages of gradually increasing running velocities at approximate intensities of 50%, 60%, 70% and 80% of VO_2max_. The same velocity (absolute intensity) was used for both the pre- and post-test. Measurements of VO_2_ and RER were made between the third and fourth minute. After the fourth minute, the mouthpiece was removed and HR was monitored until the end of the stage. Between each stage, the subjects were given one minute rest for measurement of lactate, as described above. The post-test was conducted at the same running velocities as the pre-test. Running economy (RE; mL·kg^−1^·km^−1^) was defined as VO_2_ divided by body mass and running speed. O_2_ pulse (mL·beat^−1^) was calculated by dividing VO_2_ (mL·min^−1^) by HR (beat·min^−1^). 

Training adaptations at the same relative intensity were evaluated by examining the running speed that elicited the VO_2_ value closest to 70% of the individual subject’s VO_2max_. This intensity was chosen because it produced the least variation in VO_2_ values. 

Repeated sprint test. After completing the submaximal treadmill test, all subjects performed a 5·60 m repeated sprint test in an indoor sports hall. The test was considered appropriate to induce the performance decrement associated with repeated sprint exercise [26]. All subjects performed a test-specific warm-up prior to the sprint test consisting of 3·60 m incremental runs. The sprints were performed with a 1 m flying start and each sprint was separated by 30 s of rest. Time was measured by photoelectric detectors (Brower Speed Trap II Timing system, Brower Timing system, Salt Lake USA). Verbal encouragement was given throughout the test.

20 m shuttle run test. The 20 m shuttle run test procedure was the same as previously described [27]. In short, subjects ran repeatedly between two lines, 20 m apart. The test started at a running speed of 8.5 km·h^−1^, which then increased by 0.5 km·h^−1^ per minute. The test was terminated when subjects failed to reach the 20 m line before the signal on two successive occasions. To stimulate competition, the subjects ran in groups.

### 2.4. Procedures

All tests were performed before and after the training interventions. The submaximal treadmill test and the repeated sprint test were performed on the same day, and only separated by the time to relocate from the laboratory to the sports hall. All other tests were separated by at least one resting day. Subjects were familiarized with testing procedures to minimize any potential learning effect. 

The data for this study were collected in relation to a larger study [28]. The study was approved by the Regional Ethics Committee of Oslo, Norway (ref. number 2010/1567-1) and was performed according to the Declaration of Helsinki. All subjects were informed about the purpose of the study and associated risks before they gave their written informed consent to participate.

A few subjects did not obtain valid results for all tests due to sickness, injury and unspecified withdrawal from the study. These subjects were excluded from both pre and post analysis for these particular tests. The number of participants for each test is stated in the captions of tables and figures.

### 2.5. Analysis 

Data are presented as group means ± SEM. All statistical analyses were performed in SPSS version 18 (SPSS inc., Chicago, IL, USA). The assumption of normality was evaluated by a Shapiro–Wilk test. Student’s paired *t*-test was used to investigate within-group differences, and a Student’s unpaired *t*-test was used to investigate between-group differences. A repeated measures ANOVA with a Greenhouse–Geisser correction was used to evaluate a potential increase in VO_2max_ as a function of the number of tests performed before the intervention. In cases where data was not normally distributed, a Wilcoxon signed-rank test was used to verify within-group differences, and a Mann–Whitney test was used to verify between-group differences. Statistical significance was accepted at the *p* < 0.05 level. 

## 3. Results

The number of females in each group was eight, while the number of males was four in CT and five in SIT. The mean age, height, weight and BMI was 25 ± 1 years, 175 ± 2 cm, 72.6 ± 3.8 kg and 23.6 ± 0.9 kg·m^−2^ in CT at the start of the intervention. In SIT, the mean age, height, weight and BMI was 25 ± 1 years, 173 ± 3 cm, 71.2 ± 4.1 kg and 24.0 ± 0.8 kg·m^−2^. There was no statistical difference between groups and these characteristics did not change during the intervention. Heart rate registrations at all training sessions confirmed that the subjects performed the training as recommended, including the sessions performed at home (19% of sessions). Three participants experienced minor injuries during the training intervention, including one injury unrelated to the intervention. All three were in the SIT group, and all managed to complete > 85% of training sessions.

**Figure 1:**
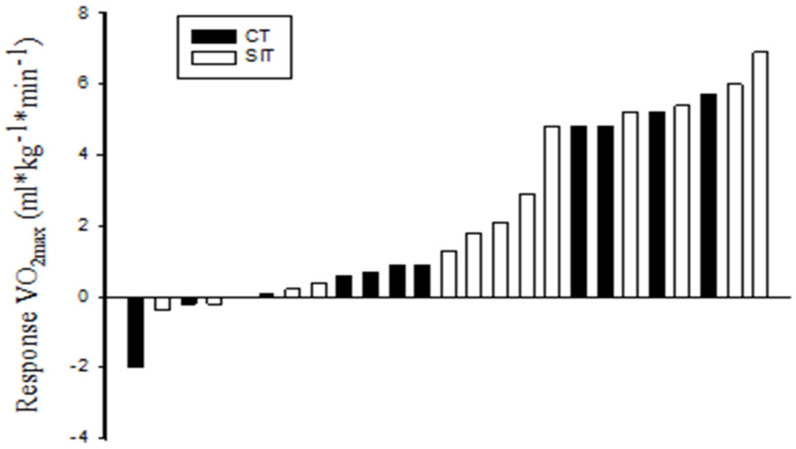
Individual change in maximal oxygen consumption (VO_2max_) after eight weeks of either continuous training (CT) or sprint interval training (SIT) (one subject in CT did not experience any change).

Maximal oxygen consumption and 20 m shuttle run performance. Maximal oxygen uptake was measured three times prior to the intervention, and VO_2max_ increased from test to test. The repeated measures ANOVA revealed that VO_2max_ increased from 48.2 ± 1.1 at the first familiarization test to 49.3 ± 1.3 in the second, and eventually to 49.9 ± 1.3 mL·kg^−1^·min^−1^ at the third test when combining both groups (*F*(1.434, 28.683) = 10.320, *p* < 0.01). VO_2max_ was improved in both CT (*p* < 0.05) and SIT (*p* < 0.01) after training (Table 1). The improvement of VO_2max_ corresponded to a 3.8% increase in CT and 5.5% in SIT. The increase in VO_2max_ varied between subjects and five subjects did not increase VO_2max_ (Figure 1). In accordance with the improved VO_2max_, both groups also increased maximal O_2_ pulse (*p* < 0.05) and the number of laps performed on the 20 m shuttle run test (CT *p* < 0.05; SIT *p* < 0.01). 

Repeated sprint test. Both the CT and SIT groups improved sprint performance for the first sprint (Table 2) and thereby improved maximal 60 m sprint performance. Both groups also improved performance on all successive sprints. However, the SIT group performed better than the CT group on sprints number four (*p* < 0.05) and five (*p* < 0.05) after the intervention (Table 2). 

Physiological response to submaximal exercise at the same absolute intensity. The submaximal treadmill test was performed at the same velocity, before and after the intervention. Both groups ran at a lower percentage of VO_2max_ after eight weeks of training (Table 3). The CT group decreased VO_2_ at all stages (i.e., running economy), while the SIT group decreased VO_2_ at stage 4 and RE at stages 2 and 4 (Table 3). HR was lower after CT at all stages (*p* < 0.01), but remained unchanged after SIT (Table 3). O_2_ pulse at submaximal intensities did not change in any group (Table 3). 

Physiological response to submaximal exercise at the same relative intensity. Adaptations to running, performed at the same relative intensity before and after the intervention, were evaluated at the velocity closest to 70% VO_2max_. At this intensity, HR remained unchanged after CT, while O_2_ pulse increased by 0.6 mL·beat^−1^ (*p* < 0.05; Table 4). In contrast, HR increased (*p* < 0.05) and O_2_ pulse remained unchanged after SIT (Table 4). RER was reduced at 70% VO_2max_ after CT (*p* < 0.05), but not statistically significant after SIT (*p* = 0.07; Table 4). Lactate was reduced at 70% of VO_2max_ in both groups after the intervention. 

## 4. Discussion

The main findings of the present study were that both training protocols increased VO_2max_ and shuttle run performance, but also produced training-specific adaptations. The SIT group performed better than the CT group on the last two 60 m sprints, while only CT improved HR and O_2_ pulse adaptations at submaximal intensities. 

The higher VO_2max_ in both groups after eight weeks of training holds implications for both performance and health, and is supported by previous research, showing a comparable improvement of VO_2max_ after sprint interval running and cycling [4,22]. Interestingly, previous research suggests that the adaptations that lead to the comparable improvement of VO_2max_ are different in the two types of training interventions. Macpherson et al. [22] showed that continuous endurance running improved maximal cardiac output, while sprint interval running did not. These reports suggest that the improvement of VO_2max_ in the present study was due to peripheral adaptations [5,6]. Importantly, the increase in VO_2max_ varied substantially between participants whether they performed CT or SIT, and five subjects did not increase their VO_2max_, even though the training was supervised by qualified instructors, and heart rate recordings confirmed that the training was performed with the recommended HR. These data agree with previous studies showing large variation in the increase in VO_2max_ after endurance training [29,30]. Genetic variation has been suggested to explain differences in the increase of VO_2max_, but research also suggests that a large number of genetic variations collectively determine increases in VO_2max_ [31]. No genetic variation predicting has so far been validated.

The increase of VO_2max_ observed after endurance exercise is caused by an improvement of cardiac output and/or arteriovenous oxygen difference [32], which results in higher VO_2_ per heartbeat (i.e., O_2_ pulse). At submaximal intensities, an increased O_2_ pulse results in lower HR [32]. In the present study, HR was reduced after CT at all submaximal velocities, while it remained unchanged after SIT. However, the participants improved running economy, which precludes the comparison of cardio-respiratory adaptations at the same absolute intensities. Therefore, to investigate the submaximal training adaptations independent of running economy, we examined HR and O_2_ pulse at the same relative intensity (~70% VO_2max_), pre and post training. At ~70% VO_2max_, O_2_ pulse increases after CT as expected (please see Table 4). In contrast, SIT did not change O_2_ pulse at ~70% VO_2max_ and HR was higher after the training intervention, supporting Macpherson et al. [22], who reported unchanged cardiac output after sprint interval running. Increased cardiac output leads to higher O_2_ pulse and decreased HR at submaximal intensities [31]. The limited cardiac adaptations after SIT in the present study suggest that CT is a superior option for cardiac adaptations, which holds implications for the health benefits of SIT, as improved cardiac function is an important part of the health benefits of exercise [33].

Several studies have shown that SIT increases the expression of oxidative enzymes in skeletal muscle [3,5,6]. In the present study, blood lactate and RER were reduced after both CT and SIT (although *p* = 0.07 for RER in SIT at 70% VO_2max_). It is well known that lactate production and RER are influenced by the oxidative capacity of skeletal muscle [34] and, thus, our results suggest that both CT and SIT improved the oxidative capacity of skeletal muscle. Results from the 20 m shuttle run test also revealed that both groups improved endurance performance and that the improvement was similar in both groups. These results are in accordance with previous investigations of both sprint interval cycling and running [3,22]. Endurance performance is a complex characteristic that is dependent on several factors, which makes it difficult to identify any single factor responsible for improved performance. Several adaptions could potentially contribute to the improved endurance performance observed in this study, but the correlation between the change of VO_2max_ and the change of 20 m shuttle run performance (r = 0.56, *p* = 0.01) suggest that VO_2max_ is central.

Results from the test of repeated sprints showed that both CT and SIT improved the performance of the first sprint and thereby improved maximal sprint performance. Improved maximal 60 m sprint performance after CT may be surprising based on the “slow paced” nature of the training intervention. However, previous research has reported similar results for untrained people, including two studies of endurance cycling reporting improved sprint performance after continuous training [34,35]. The mechanisms behind these improvements are uncertain, but mechanical efficiency has been suggested as the most important factor [35]. In the present study, CT improved RE, which is a common measure of mechanical efficiency [36]. Improved RE could therefore offer an explanation for improved maximal sprint performance after CT. Improvements of mechanical efficiency is often associated with increased stiffness of muscles and tendons, but improved running technique by wasting less energy on braking forces and excessive vertical oscillation may be a likely cause for the improvement in CT [36], since the participants were inexperienced runners with a high potential for improving running technique. Improved maximal running velocity after SIT has previously been reported [22], and is supported by findings of improved peak power after sprint interval cycling [4,8,9,19].

Both groups also improved repeated sprint ability. These results are in accordance with previous studies that have investigated RSA after continuous training and high intensity interval training [34,35,37]. Interestingly, the SIT group performed better than the CT group on sprints number four and five after the intervention, thus demonstrating a superior ability to resist fatigue. The reason for the improved performance on the last two sprints could be related to the ability of SIT to increase muscle buffer capacity and levels of anaerobic enzymes [3,6], and to prevent metabolic and ionic perturbation during high-intensity exercise [8]. All of these adaptations can potentially improve performance during repeated sprint exercise [26]. The benefit of improved buffer capacity and ability to prevent ionic and metabolic perturbations would be progressively more beneficial during repeated sprint exercise, which may explain why SIT performed better at sprint number 4 and 5, and not 1, 2 and 3.

Some limitations in the present study need to be recognized. The number of participants included in this study was based on an a priori power analysis for the between group comparison of VO_2max_. However, the statistical power may still be limited for the other comparisons in this study, in particular for tests with missing data. The results at the 70% intensity should be considered carefully. As explained in the methods, running speed was not adjusted to exactly 70% VO_2max_ and there was some individual variation in running intensity from pre- to post-test. However, these variations were small, and mean relative intensity varied by less than one percentage-point between pre- and post-test (Table 4). The majority of participants were female, and although training groups were gender matched, we did not control for oral contraceptive use and menstrual cycle phase. Furthermore, high intensity exercise is commonly associated with increased risk of injury [38], and in the present study, we were unable to prevent the occurrence of injuries in the SIT group, despite a standardized warm-up and supervision of highly qualified personnel. Strengths of this study include the fact that heart rate was recorded at all training sessions, and that both females and males were included. Furthermore, a substantial effort was made during familiarization, to reduce the potential impact of a learning effect on VO_2max_ from pre- to post-test. VO_2max_ did increase during the familiarization process, but levelled off from the last familiarization test to the pre-test, which indicates that the efforts was successful at minimizing the learning effect in this study. However, the inclusion of the familiarization tests in addition to an already high number of pre-tests may have resulted in some minor training adaptations before the onset of the training interventions.

## 5. Conclusions

In conclusion, both types of training produced similar improvements of VO_2max_, endurance capacity and sprint performance. Despite these similarities, O_2_ pulse and HR during submaximal exercise was improved after CT only, which suggests superior adaptations of cardiac health after CT compared to SIT. In addition, SIT improved RSA significantly more compared to CT. The present study therefore suggest that training-specific adaptations occur after sprint interval running and continuous running with moderate intensity. The presumption of training-specific adaptations should be taken into consideration when composing an optimal endurance training program.

## Figures and Tables

**Table 1:** Parameters of maximal endurance performance before and after eight weeks of continuous training (CT) and sprint interval training (SIT).

	CT		SIT	
	Pre	Post	Pre	Post
VO_2max_ (mL·kg^−1^·min^−1^)	47.9 ± 1.5	49.7 ± 1.5 *	50.5 ± 1.6	53.3 ± 1.5 *
Maximal O_2_ pulse	17.4 ± 1.0	18.1 ± 1.0 *	18.0 ± 1.0	19.2 ± 1.0 *
Laps	71.5 ± 6.1	79.4 ± 5.2 *	69.5 ± 3.8	81.7 ± 4.0 *

Values are mean ± SEM. CT, *n* = 12 (4 males, 8 females). SIT, *n* = 13 (5 males, 8 females). VO_2max_, maximal oxygen consumption; O_2_ pulse, oxygen pulse; Laps, number of laps completed during the 20 m shuttle run test. * Statistically significant difference from pre (student’s t-test), *p* < 0.05. There were no statistically significant differences between groups.

**Table 2:** Performance on the repeated sprint test before and after eight weeks of continuous training (CT) and sprint interval training (SIT).

	CT				SIT			
	Pre		Post		Pre		Post	
	Time (s)	%dec.	Time (s)	%dec	Time (s)	%dec	Time (s)	%dec
1. 60 m	9.92 ± 0.25		9.69 ± 0.26 *		9.64 ± 0.26		9.20 ± 0.21 *	
2. 60 m	10.44 ± 0.33	5.2	10.06 ± 0.27 *	3.8	9.98 ± 0.23	3.5	9.48 ± 0.18 *	3.0
3. 60 m	10.76 ± 0.29	8.5	10.31 ± 0.23 *	6.4	10.27 ± 0.22	6.5	9.89 ± 0.20 *	7.5
4. 60 m	10.87 ± 0.30	9.6	10.54 ± 0.23 *	8.8	10.37 ± 0.25	7.6	9.91 ± 0.19 *^,†^	7.7
5. 60 m	10.93 ± 0.21	10.2	10.70 ± 0.22 *	10.4	10.53 ± 0.25	9.2	9.96 ± 0.20 *^,^^†^	8.3

Values are mean ± SEM. CT, *n* = 10. SIT, *n* = 11. %dec = percent performance decrement compared to the fastest sprint time * Statistically significant difference from pre.^†^ Statistically significant difference from CT (student’s t-test), *p* < 0.05.

**Table 3:** Physiological responses at submaximal velocities, before (pre) and after (post) eight weeks of either continuous training (CT) or sprint interval training (SIT).

		1		2		3		4	
		Pre	Post	Pre	Post	Pre	Post	Pre	Post
**CT**	VO_2_ (mL·min^−1^)	1553 ± 139	1381 ± 159 *^,#^	2307 ± 177	1876 ± 157 *	2414 ± 175	2275 ± 174 *	2754 ± 186	2620 ± 172 *
	% VO_2max_	45.1 ± 3.4	37.8 ± 2.1 *	58.4 ± 3.1	52.4 ± 2.8 *	71.1 ± 2.3	64.6 ± 2.4 *	79.4 ± 2.0	73.3 ± 1.8 *
	RE (mL·kg^−1^·km^−1^)	213 ± 16	186 ± 10 *^,#^	229 ± 12	213 ± 10 *	238 ± 8	224 ± 8 *	232 ± 6	223 ± 6 *
	% HR_peak_	66.9 ± 2.1	59.6 ± 2.3 *	76.8 ± 2.2	70.8 ± 2.5 *	85.3 ± 1.5	80.2 ± 2.1 *	90.0 ± 1.0	86.3 ± 1.4 *
	O_2_pulse (mL·beat^−1^)	11.5 ± 0.7	11.5 ± 1.0	13.1 ± 0.8	13.3 ± 0.9	14.2 ± 0.8	14.1 ± 0.9	15.3 ± 0.9	15.2 ± 0.9
	RER (VCO_2_·VO_2_^−1^)	0.89 ± 0.01	0.84 ± 0.01 *	0.93 ± 0.01	0.88 ± 0.01 *	0.94 ± 0.01	0.90 ± 0.01 *	0.97 ± 0.01	0.93 ± 0.01 *
	Lactate (mmol·l^−1^)	1.22 ± 0.13	0.77 ± 0.06 *	1.76 ± 0.26	1.16 ± 0.13 *	2.39 ± 0.25	1.75 ± 0.17 *	3.84 ± 0.30	2.66 ± 0.24 *
**SIT**	VO_2_ (mL·min^−1^)	1544 ± 152	1523 ± 150	2221 ± 168	2076 ± 158	2574 ± 181	2500 ± 165	2909 ± 204	2832 ± 196 *^,#^
	% VO_2max_	42.6 ± 2.2	40.1 ± 2.5 *	61.9 ± 1.6	55.3 ± 2.0 *	71.8 ± 1.2	66.5 ± 1.5 *	81.2 ± 0.9	75.2 ± 1.2 *
	RE (mL·kg^−1^·km^−1^)	201 ± 10	199 ± 10	243 ± 8	228 ± 8 *	240 ± 5	234 ± 4	237 ± 4	231 ± 4 *
	% HR_peak_	61.6 ± 2.4	61.6 ± 2.5	75.0 ± 1.6	72.0 ± 2.0	82.6 ± 1.2	81.1 ± 1.5	88.6 ± 0.9	87.2 ± 1.2
	O_2_pulse (mL·beat^−1^)	12.8 ± 1.0	12.6 ± 1.0	14.9 ± 1.0	14.5 ± 1.1	15.8 ± 1.0	15.6 ± 0.9	16.6 ± 1.0	16.4 ± 1.0
	RER (VCO_2_·VO_2_^−1^)	0.86 ± 0.02	0.83 ± 0.02	0.91 ± 0.01	0.87 ± 0.02 *^,#^	0.92 ± 0.01	0.89 ± 0.01 *	0.96 ± 0.01	0.92 ± 0.01 *
	Lactate (mmol·l^−1^)	1.12 ± 0.08	0.89 ± 0.06 *	1.79 ± 0.15	1.20 ± 0.07 *^,#^	2.38 ± 0.17	1.68 ± 0.12 *	3.45 ± 0.24	2.66 ± 0.19 *^,#^

Values are mean ± SEM. VO_2_, oxygen consumption; % VO_2max_, percent of maximal oxygen consumption; RE, running economy; % HF_peak_, percent of peak heart rate; O_2_ pulse, oxygen pulse; RER, respiratory exchange ratio. Velocities at stage 1, 2, 3 and 4 equaled 6.2 ± 0.2, 7.5 ± 0.2, 8.8 ± 0.3 and 10.1 ± 0.3 km·h^−1^ in CT, and 6.4 ± 0.2, 7.7 ± 0.2, 9.1 ± 0.3 and 10.4 ± 0.3 km·h^−1^ in SIT. CT, *n* = 11. SIT, *n* = 13. Values for % HF_peak_ and O_2_ pulse represents only 12 subjects in SIT. * Statistically significant difference from pre (student’s t-test), *p* < 0.05. ^#^ Statistically significant difference from pre (verified by Wilcoxon signed rank test), *p* < 0.05. There were no statistically significant differences between groups.

**Table 4:** Physiological responses to running at the velocity closest to 70 percent of maximal oxygen consumption, before (pre) and after (post) eight weeks of either continuous training (CT) or sprint interval training (SIT).

	CT		SIT	
	Pre	Post	Pre	Post
Velocity (km·h^−1^)	8.7 ± 0.4	9.7 ± 0.3 *	8.8 ± 0.4	9.6 ± 0.3 *
% VO_2max_	70.5 ± 0.9	71.4 ± 0.7	70.3 ± 0.7	70.2 ± 0.6
% HR_peak_	84.3 ± 1.0	85.1 ± 0.9	81.9 ± 1.6	84.9 ± 1.4 *
O_2_ pulse (mL·beat^−1^)	14.5 ± 0.9	15.1 ± 0.9 *	15.7 ± 1.0	16.0 ± 0.9
RER (VCO_2_·VO_2_^−1^)	0.94 ± 0.01	0.91 ± 0.01 *	0.93 ± 0.01	0.91 ± 0.01
Lactate (mmol·l^−1^)	2.62 ± 0.16	2.18 ± 0.19 *^,#^	2.33 ± 0.12	2.03 ± 0.15 *^,#^

Values are mean ± SEM. % VO_2max_, percent of maximal oxygen consumption; % HF_peak_, percent of peak heart rate; O_2_ pulse, oxygen pulse; RER, respiratory exchange ratio. CT, *n* = 11. SIT, *n* = 13. Values for % HF_peak_ and O_2_ pulse represents only 12 subjects in SIT. * Statistically significant difference from pre (student’s t-test), *p* < 0.05. ^#^ Statistically significant difference from pre (verified by Wilcoxon signed rank test), *p* < 0.05. There were no statistically significant differences between groups.
